# Quality of diabetes mellitus healthcare and metabolic control during transition from paediatric to adult care: A systematic review and meta‐analysis

**DOI:** 10.1111/dme.70125

**Published:** 2025-08-20

**Authors:** Giovanna Donvito, Carlo Maltecca, Sabine E. Hofer, Dagmar Meraner, Uwe Siebert, Marjan Arvandi

**Affiliations:** ^1^ Institute of Public Health, Medical Decision Making and Health Technology Assessment, Department of Public Health, Health Services Research and Health Technology Assessment UMIT TIROL—University for Health Sciences and Technology Hall in Tirol Austria; ^2^ Department of Medicine and Surgery University of Milano‐Bicocca Milan Italy; ^3^ Department of Child and Adolescent Psychiatry University of Freiburg Freiburg Germany; ^4^ Department of Paediatrics 1 Medical University of Innsbruck Innsbruck Austria; ^5^ Center for Health Decision Science, Department of Epidemiology Harvard T.H. Chan School of Public Health Boston Massachusetts USA; ^6^ Center for Health Decision Science, Department of Health Policy & Management Harvard T.H. Chan School of Public Health Boston Massachusetts USA; ^7^ Program on Cardiovascular Research, Institute for Technology Assessment and Department of Radiology Massachusetts General Hospital, Harvard Medical School Boston Massachusetts USA

**Keywords:** adult care, diabetes, diabetes healthcare, HbA_1c_, paediatric diabetes, paediatric healthcare, transition

## Abstract

**Aims:**

Emerging adults with chronic diseases like diabetes often experience a decline in health during the process of transition from paediatric to adult healthcare. This study assesses the impact of transition on healthcare quality of people with diabetes, focusing on glycated haemoglobin (HbA_1c_).

**Methods:**

We conducted a systematic review and meta‐analysis of the difference in HbA_1c_ before and after transition following the PRISMA guidelines. A comprehensive search across four databases for studies of diabetes type 1 and 2 published between 2018 and 2024 was conducted. Risk of bias was assessed using the ROBINS‐I tool for non‐randomized studies.

**Results:**

Twenty studies were included in the systematic review, fifteen to the meta‐analysis. Eleven studies considered a structured or semi‐structured transition programme: three reported a reduction in HbA_1c_ (improved glycaemic control) and eight no significant difference. Nine studies considered no transition programme: four showed a worsening of glycaemic control and five no significant difference. Overall, the meta‐analysis showed a worsening of glycaemic control post‐transition with mean difference (MD) −1.75 mmol/mol (−0.16%) [95% confidence interval (CI) –5.24–1.75 mmol/mol (−0.48%–0.16%)], with considerable heterogeneity, where negative values indicate higher HbA_1c_ post‐transition. Subgroup analysis for transition programmes and usual care showed, respectively, significant improvement and worsening of glycaemic control with MD of 3.28 mmol/mol (0.30%) [95% CI 0.44–6.12 mmol/mol (0.04%–0.56%)] and −6.99 mmol/mol (−0.64%) [95% CI −11.79 to −2.19 mmol/mol (−1.08% to −0.20%)].

**Conclusions:**

Findings suggest that the transition to adult care may negatively affect glycaemic control in emerging adults with diabetes, whereas structured transition programmes can neutralize this effect and prevent consequences. Further investigations are needed to develop evidence‐based guidelines for optimizing transition interventions.

## Graphical abstract

Transition programs may help maintain or improve glycaemic control during transition from paediatric to adult healthcare in adolescents and young adults with diabetes type 1 and 2.Note: HbA1c values are reported here in percentage (%) according to the Diabetes Control and Complications Trial (DCCT) units for clarity of the figure. These values can be converted to International Federation of Clinical Chemistry and Laboratory Medicine (IFCC) units (mmol/mol) using the official IFCC conversion tables

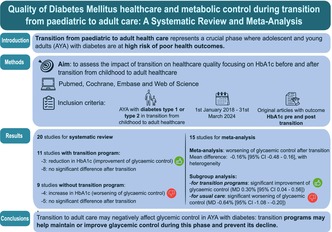

**What's new?**

**What is already known?**

- Given the challenges emerging adults with diabetes face during the transition from paediatric to adult healthcare, we aimed to assess the impact of this phase and of transition programs on glycaemic control, focusing on changes in glycated haemoglobin levels in adolescents and young adults (AYA) with type 1 and type 2 diabetes.

**What did we find?**

- Transition to adult care generally worsened glycaemic control; however, transition programs helped to mitigate this, showing improved glycaemic control compared with usual care.

**What are the implications?**

- These findings highlight the importance of transition programmes in maintaining or improving glycaemic control during this vulnerable life phase.

## 1 INTRODUCTION

Diabetes mellitus is a metabolic disease, characterized by chronic hyperglycaemia and classified in autoimmune type 1 diabetes and type 2 diabetes resulting from genetic, environmental and metabolic causes that differ among populations.^1^

Current evidence suggests that diabetes prevalence is continuously rising worldwide, posing a major challenge to global health.^1^, ^2^ This rapidly increasing incidence represents a significant problem not only in adults but also in the paediatric and young populations.^2^

Chronic hyperglycaemia can lead to severe long‐term consequences such as microvascular and macrovascular complications, which may result in dysfunction and failure in the nervous system, kidneys, eyes and blood vessels, increasing the risk of cardiovascular diseases.^1^ Achieving optimal glycaemic control, commonly assessed through glycated haemoglobin (HbA_1c_) measurements in blood, is therefore crucial to minimise these risks. Current guidelines recommend HbA_1c_ targets ≤6.5% for youth with access to advanced diabetes technologies^3^, ^4^; however, this was not the standard target in many of the studies included in this review.

Numerous studies^5^, ^6^ have documented that the process of transition from paediatric to adult healthcare represents a crucial and vulnerable phase where young adults with chronic diseases, such as diabetes, are at high risk of poor health outcomes, both during this process and afterwards. Adolescents and young adults (AYA) with type 1 or type 2 diabetes face considerable challenges in maintaining stable metabolic control due to hormonal fluctuations, psychosocial stressors and the increased responsibility associated with autonomous disease management.^6^ Elevated HbA_1c_ levels are frequently observed in this age group and are strongly linked to a heightened risk of acute complications, such as diabetic ketoacidosis, as well as early onset of long‐term complications.^3^ Moreover, this developmental period coincides with significant life transitions, such as completing education, entering the workforce and adapting to evolving social and family dynamics, which may further disrupt effective diabetes self management.^7^

Evidence indicates that suboptimal transition processes are associated with gaps in care, deteriorating glycaemic control, and increased rates of acute complications and hospitalisations.^8^ Consequently, there is growing consensus regarding the necessity for structured, evidence‐based transition programmes that address the complex medical, psychological and social needs of adolescents and young adults with diabetes.^9^

Guidance from GotTransition.org^10^ recommends initiating transition planning as early as ages 12 to 14, emphasising that readiness should be based on individual maturity rather than chronological age. Essential elements include preparing a comprehensive written health summary for the adult care team, systematic education in self management skills and proactive coordination between paediatric and adult care providers to ensure continuity of care.^10^ Complementing these recommendations, the American Diabetes Association highlights that emerging adulthood frequently coincides with major life changes and stresses the importance of addressing psychosocial factors, conducting routine screening of metabolic and mental health parameters and empowering young adults to take independent responsibility for their diabetes management.^11^

Therefore, it is essential to systematically explore the current literature on the impact of transition on healthcare outcomes, particularly HbA_1c_ levels, to provide evidence for public health regulators. This evidence should support the development of clinical guidelines and best practices to ensure better patient support and improve glycaemic control during this critical period of transition for AYA with diabetes.

Given recent findings in this area^12^, ^13^ and the findings of a previous meta‐analysis by Schultz and Smaldone^5^ in 2017, showing no significant improvement in HbA_1c_ after transition between the intervention and control group but a significant reduction of diabetic ketoacidosis in the intervention group, it is of utmost importance to integrate and evaluate them in a timely manner. Our study builds on this by including more recent studies and focusing on pre‐ and post‐transition HbA_1c_ outcomes. A recent systematic review^14^ found no clear metabolic benefit for transition interventions to improve transition readiness and/or transfer to adult care, highlighting the need for further exploration. Therefore, the goal of this research is to conduct a systematic review and meta‐analysis, to synthesize the most recent and relevant evidence, assessing the impact of the transition, both with and without structured programs, on healthcare outcomes for AYA with diabetes, specifically regarding metabolic control and HbA_1c_ levels.

## 2 METHODS

### 2.1 Literature search and inclusion criteria

The present systematic review and meta‐analysis was conducted in accordance with the Preferred Reporting Items for Systematic Reviews and Meta‐Analyses (PRISMA 2020) checklist.^15^

A comprehensive literature search was performed across four databases: PubMed, Cochrane, Embase and Web of Science covering publications between 2018 and 31 March 2024. The search strategy employed a combination of search terms and Boolean operators, including “diabetes”, “healthcare”, “transition of care”, “paediatrics”, “children”, “adult”, combined with “AND” and “OR” operators.

Inclusion and exclusion criteria were developed based on the PICO framework (participants, interventions, control, outcomes), which is summarized in Table S1. We included studies that met the following criteria: (i) studies published in English, German, Italian, Spanish or French; (ii) investigating people with any diabetes type undergoing transition of healthcare from childhood to adult healthcare; (iii) published not before 2018; and (iv) investigating HbA_1c_ as a healthcare outcome, reporting HbA_1c_ data both before and after the transfer to adult healthcare. The following study types were excluded: guidelines, meta‐analyses, reviews, letters, comments, posters, editorials and conference abstracts. By adhering to these criteria, we aimed to ensure the inclusion of relevant and high‐quality studies that specifically addressed the impact of healthcare transition on HbA_1c_ levels in AYA with diabetes transitioning from paediatric to adult care, focusing on the most recent publications to better reflect current evidence and evolving practices in transitional care.

### 2.2 Data extraction

Records retrieved through our search were imported into Zotero, where duplicates were removed. After deleting duplicate studies and applying the selection criteria, two independent and blinded reviewers screened the titles and abstracts and selected reports for full‐text review. Possible disagreements were resolved by a third reviewer and discussed until reaching a consensus. To represent this process through a flow diagram, we used the online tool as recommended by the PRISMA guidelines.^15^

The primary outcome assessed was the change in HbA_1c_ levels before and after the transition from paediatric to adult healthcare providers.

Data extracted from each study included: sample size, study location, year of publication, study duration, age at transition, sex, components of the transition program and study outcomes. All extracted data were compiled into an Excel spreadsheet and combined across studies to provide a comprehensive analysis of the transition's impact on HbA_1c_ levels.

### 2.3 Quality assessment

To assess the quality of the studies included in this review, we used the Risk Of Bias In Non‐randomized Studies of Interventions (ROBINS‐I) tool, designed for evaluating the risk of bias in non‐randomized studies of interventions (including uncontrolled before‐after studies).^16^ We used the ROBINS‐I tool because the majority of included studies investigated interventions in a non‐randomized design, and ROBINS‐I is a widely recommended and structured tool. To facilitate this assessment and to visually summarize the results we used an online platform.^17^ For each of the seven domains of bias^16^ assessed through the ROBINS‐I tool, studies were evaluated and categorized as having a critical, serious, moderate or low risk of bias, or as having no information available.

### 2.4 Statistical analysis

Studies that clearly reported mean HbA_1c_ values pre‐ and post‐transition along with their standard deviation were eligible for inclusion in the meta‐analysis to estimate the pooled mean difference and the corresponding standard deviation. HbA_1c_ values that were reported in International Federation of Clinical Chemistry units (mmol/mol) were converted to National Glycohemoglobin Standardization Program units (%).^18^ For studies containing only median and interquartile range for the HbA_1c_ value, we converted it to the mean value with standard deviation using a standardized methodology according to Hozo et al.^19^ Given the expected heterogeneity across studies, we a priori selected the random effects model (REM) to estimate the mean difference in HbA_1c_ pre‐ and post‐transition. The *I* ^2^ statistic and the Q test were used to quantify heterogeneity between studies. Furthermore, subgroup analyses were carried out in order to pinpoint the cause of heterogeneity. Through the use of Egger's regression test^20^ and funnel plot inspection, potential publication bias was visually evaluated. *p*‐values were considered significant at *α* < 0.05.

Statistical analysis was performed using R software version 4.3.2 (R Core Team, Vienna, Austria), using package ‘meta’.^21^

## 3 RESULTS

**FIGURE 1:**
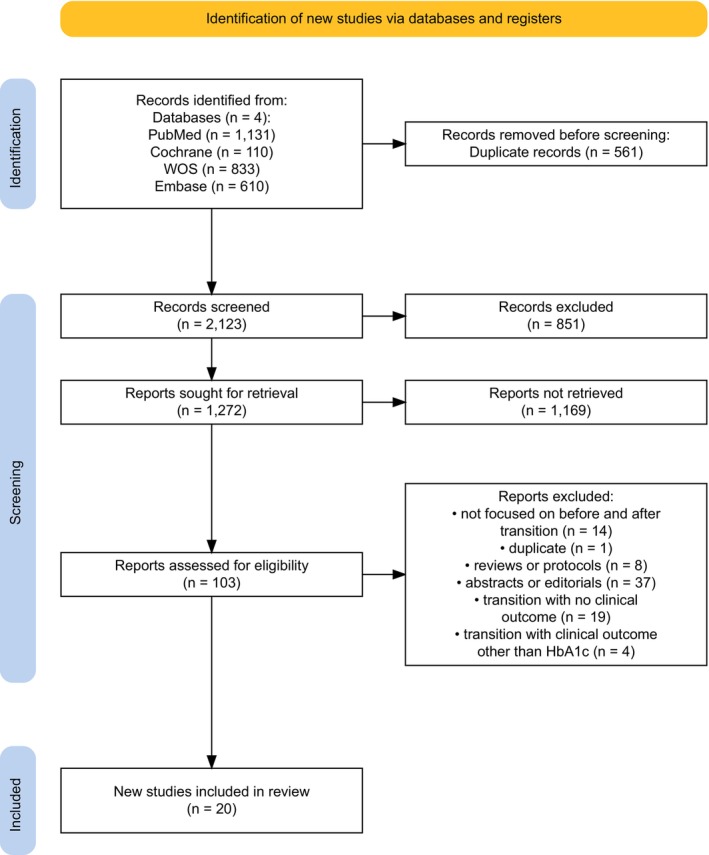
PRISMA flow diagram of study identification and selection.

As shown in Figure 1, a total of 2684 articles were identified across four databases. After removing 561 duplicates and excluding 851 articles published before 2018, 1272 records were screened by title and abstract. Of these, 123 full‐text articles were assessed for eligibility. Disagreements between the two reviewers, resolved by a third reviewer, resulted in 103 articles retained for full‐text evaluation. Following full‐text assessment, twenty studies were included in the systematic review. The remaining 83 articles were excluded for the following reasons: lack of pre‐ and post‐transition data (*n* = 14), duplicates (*n* = 1), non‐original research (guidelines, reviews or protocols; *n* = 8), abstracts or commentaries (*n* = 37), no clinical outcomes reported (*n* = 19) or outcomes other than HbA_1c_ (*n* = 4). Of the twenty included studies, fifteen reported the mean and standard deviation of HbA_1c_ and were therefore eligible for inclusion in the meta‐analysis.

The final twenty studies for the systematic review included studies of various study designs, including cohort studies, retrospective studies, as well as non‐randomized clinical trials.

### 3.1 Studies characteristics

Table 1 shows an overview of the studies included in this review and fully describes their characteristics. As shown, eight studies were conducted in the United States, two in Canada, six in Europe, one in Tunisia and three in Australia. The majority (75%) of the studies focused exclusively on type 1 diabetes, while three studies dealt with both types, and two considered only type 2 diabetes in adolescents and young adults (AYA). Eleven studies evaluated a structured or semi‐structured transition programme, and five out of the twenty studies also accounted for other factors, such as mental health conditions, healthcare coverage degree, hypoglycaemia unawareness, the co‐location of paediatric and transition clinics and the duration of the transition period from childhood to adult healthcare.

**TABLE 1:** Overview of study designs and key features.

Author, year, country and study design	Diabetes type	Study population size	Women, *n* (%)	Mean age at transfer, years (SD or min‐max)	Transition intervention/Program Description	Mean HbA_1c_ (SD or min‐max) before and after	HbA_1c_ variations—conclusions	Other characteristics (or RF) associated to the transition
Iyengar et al.,^31^ 2024, USA, single‐centre cohort study	1 and 2	Type 1 diabetes: 300 (256 with both baseline and post‐transfer HbA_1c_); Type 2 diabetes: 16 (13 with both baseline and post‐transfer Hb A_1c_)	–	Type 1 diabetes: 20.9 (1.5); Type 2 diabetes: 20.8	Structured transition program with care coordination led by the adult endocrinology with key elements such as assessment of transition readiness and transition coordinator	Type 1 diabetes: HbA_1c_ at or <3 months before the initial adult visit 9.1 ± 2.1%, HbA_1c_ at 12 months after care transfer 8.7 ± 1.9% Type 2 diabetes: HbA_1c_ at or <3 months before the initial adult visit 8.6 ± 2.2%, HbA_1c_ at 12 months after care transfer 7.6 ± 2.3%	Type 1 diabetes: where both baseline and post‐transfer HbA_1c_ were available modest but significant improvement observed (baseline mean 9.1 ± 2.1% and post‐transfer mean 8.7 ± 1.9%, *p* < 0.01 by paired *t* test). Type 2 diabetes: Among participants for whom both baseline and 1‐year post‐transition HbA_1c_ were available, there was a non‐significant trend toward improved glycaemic control from 8.2% to 7.6% (*p* = 0.23, paired *t* test)	–
Hodnekvam et al.,^36^ 2023, Norway, population‐based cohort study (questionnaire)	1	321	185 (57.6)	18.0 (15.0–23.5)	–	HbA_1c_ at the last registration: 8.8% (5.5%–15.8%)	Group in the intermediate‐decrease trajectory (*n* = 218): descending from mean 8.3% at 14 y to 7.5% at 24. Group of the high‐arch trajectory (*n* = 102), starting at mean 9.4%, increased to higher than 9.9% between 16 and 21 y, decreased to 9.0% at 24 y	–
Schweizer et al.,^12^ 2023, Germany, Retrospective, follow‐up study (questionnaire)	1	190	91 (47.9)	21.2 (2.5)	Semi‐structured program for transfer: initial consultation with an adult diabetologist prior to transition	Baseline: 7.6% (1.1); no clear data about follow‐up on tables but on a figure ‘With increasing time after transfer and thus increasing age, the HbA_1c_ level decreases’	No difference in HbA_1c_ levels between people in f‐up by resident diabetologists and in f‐up in diabetes‐specific outpatient clinics. People not in diabetes‐specific care shown significantly higher HbA_1c_ levels than the others (7.5 ± 1.1% vs. 7.1 ± 0.8%)	–
Harmer et al.,^22^ 2022, UK, Single‐centre retrospective observational study	1	106 (35 in non‐RSG; 71 in RSG)	18 (51.4) in non‐RSG; 40 (56.3) in RSG	Median: 18 (18–25)	‘Ready Steady Go’ (RSG) holistic structured transition program	Non‐RSG group: pre‐transfer 10.1% (5.8); post‐transfer 10.9% (6.1); RSG group: pre‐transfer 9.2% (5.2); post‐transfer 9.3% (4.9)	In the intervention group, no rise in HbA_1c_ usually observed in young people during early adult life	–
Peeters et al.,^23^ 2022, Netherlands, retrospective chart review	1	320 (130 LO‐ATT; 190 HI‐ATT)	64 (49.2) in LO‐ATT; 82 (43.2) in HI‐ATT	18.64 (1.77) in LO‐ATT; 18.62 (1.11) in HI‐ATT	More (HI‐ATT) versus less attention (LO‐ATT) to transitional care	Paediatric (*n* = 261): 8.7 (1.33); adult care (*n* = 261): 8.6 (1.32). LO‐ATT (*n* = 91): 8.6% (71; ±13.57); HI‐ATT (*n* = 141) 8.6% (70; ±15.34)	No appreciable variations in HbA_1c_ between adult and paediatric care. With the exception of transfer readiness, the Dutch diabetes care system's current transitional care investments have not significantly improved experiences or results	–
Soliman et al.,^37^ 2022, USA, retrospective chart review	1	214	116 (54.2)	Median (Q1–Q3): 21.5 (19.9–23.1)	–	Last paediatric HbA_1c_ (*n* = 200): 8.73 (5.00–15.10)	Transitioning at an older age was associated with a lower rate of hospitalization and ED visits but not significantly associated with HbA_1c_	–
Tilden et al.,^38^ 2022, USA, Single‐centre retrospective cohort study	1	449	271 (48.3)	19.8 (1.3)	–	Mean HbA_1c_ 1 y before transfer: 9.0% (75 mmol/mol; SD 1.9%); Mean HbA_1c_ 1 y after transfer: 8.9% (74 mmol/mol; SD 2.0%)	An increase in average HbA_1c_ of 0.31% (3 mmol/mol; 95 %CI 0.09%–0.53%) for every 6 months of delay between the last paediatric and first adult visits (unadjusted model); after adjustment: 0.19% (2 mmol/mol; 95 %CI 0.04%–0.33%)	Prolonged lapses between paediatric and adult care
Ali et al.,^39^ 2021, Australia, retrospective medical records review	1	356 (121: internally referred from the hospital's paediatric clinic (IRG); 235: externally referred from other sources paediatric(ERG))	54 (44.6) in IRG; 117 (49.8) in ERG	17.1 ± 1.7 in IRG; 18.8 ± 2.6 in ERG	–	IRG: mean HbA_1c_ pretransition 9.1% ± 1.9%, at 6 months 9.2%, at 12 months 9.0%; ERG: mean HbA_1c_ pretransition vs. 9.7% ± 2.6%, at 6 months 8.4%, at 12 months 8.3%	Improvements in HbA_1c_ were seen only in the ERG at 6 and 12 months (*p* < 0.001). Although co‐location of a paediatric and transition clinic improved medical engagement, this did not equate to better glycaemic control or complication rates	Co‐location of a paediatric and transition clinic
Butalia et al.,^24^ 2021, Canada, Non‐randomized clinical trial	1	203 (102 usual care; 101 intervention)	53 (52.0) in usual care; 48 (47.5) in intervention	18.6 (0.4) in usual care; 18 (0.4) in intervention	*Control group*: usual care; *Intervention group*: received usual care + support from a transition coordinator (using communication technology)	1 year prior to transfer in usual care: 9.3% (1.8); in intervention 8.9% (2.0) 1 year after transfer in usual care 9.2% (1.8); in intervention 8.8% (1.9)	No significant differences in HbA_1c_ post‐transfer	–
Sritharan et al.,^25^ 2021, Australia, Retrospective study (audit)	1, 2 and other	220	92 (44.5)	19.2 (1.8)	Diabetes transition clinic: a multidisciplinary (endocrinologist, educator, dietitian) Clinic with one of the educators serving as a coordinator	At entry: 9.9% (2.6); At last visit: 8.9% (2.3)	Improvement in glycaemic control at last visit in comparison with entry values. Over eight years, those with, compared with those without mental health conditions had higher HbA_1c_ at the last visit (9.4% (79 mmol/mol) vs. 8.7% (71 mmol/mol), *p* = 0.027)	Associated mental health conditions
TODAY Study Group,^32^ 2020, USA, Observational follow‐up study	2	421 in 2013; 427 in 2016	63 (15.0) in 2013; 64 (15.0) in 2016	21 (3) in 2013; 24 (2) in 2016	–	In 2013: 8.8% (2, 9); In 2016: 9.6% (3.0)	Comparing healthcare coverage and glycaemic control during the 2 years before transition to community care (TODAY2 phase 1, when participants received diabetes care from the study team at no cost) to the 2 years after the transition to community care in the TODAY2 cohort, glycaemic control remained poor, regardless of coverage	Healthcare coverage: government, commercial, no coverage
Vidal et al.,^29^ 2020, Spain, Retrospective observational study	1	56	26 (46.4)	18.1 ± 0.3	Therapeutic care and education program in 4 phases (12 months): a transfer process coordinated from paediatric and adult units, and individual, group and telematic visits	At baseline (at transition moment): 8.01% (1.22); after transition (at 12 months): 7.70% (0.99)	Non‐significant reductions were observed in HbA_1c_	Additional evaluation of hypoglycaemia unawareness (HU): TEP improves HU but does not solve the problem
Walch et al.,^40^ 2020, USA, Retrospective chart review	1	54	26 (48.1)	18.1 (1.0)	–	Before transition: 9.0%; after transition: 9.2%	The HbA_1c_ was stable over the transition period	–
Spaic et al.,^26^ 2019, Canada, Multi‐centre, randomized, parallel‐group, controlled trial	1	104 in structured transition program; 101 in standard care	47 (45.2) in intervention; 54 (53.5) in usual care	17.9 (0.7) in intervention group; 17.9 (0.6) in standard group	Standard care vs. Transition program (with a transition coordinator). The intervention lasted 18 months (6 in paediatric and 12 in adult care)	Intervention group: Baseline (*n* = 103) 8.46% (1.30); Intervention period (0–18 months; *n* = 63) 8.59% (1.47); F‐up period (18–24 months, *n* = 73) 8.33% (1.21); Standard group: Baseline (*n* = 99) 8.61% (1.57); Intervention period (*n* = 50) 8.63% (1.49); F‐up period (*n* = 71) 8.80% (1.55)	Intervention group: Change from baseline to intervention period in HbA_1c_: −0.20% (1.24); Change from baseline to f‐up period in HbA_1c_: 0.03% (1.09) → this positive difference means a decrease (improvement) from the baseline HbA_1c_‐value; Standard group: Change from baseline to intervention period in HbA_1c_: −0.19% (1.29); Change from baseline to f‐up period in HbA_1c_: −0.28% (1.64). Trend toward improvement in average HbA_1c_ (8.33% vs. 8.80%), although during the f‐up, the mean change in HbA_1c_ did not differ between groups	
Agarwal et al.,^33^ 2018, USA, Multi‐centre, follow‐up study	2	182	117 (64.3)	Up to 25 y	–	Baseline: 7.0%; follow‐up: in adult care: 9.0%; no care: 9.3 paediatric care group: 8.0%	Transferring from paediatric care was associated with a 4.5 and 4.6 higher odds of poor glycaemic control at f‐up in adult and no care, respectively, after adjustment	–
Berg et al.,^34^ 2018, USA, Longitudinal, multisite study (survey)	1	220	138 (63.0)	17.77 (0.39)	–	Baseline: 8.218% (1.668); Year 2: 8.937% (1.976); Year 3: 9.229% (2.052)	HbA_1c_ increased significantly across the three time points	–
Essaddam et al.,^27^ 2018, Tunisia, Longitudinal study	1	48	25 (52.1)	16.3 (1.56)	Structured program of transition: transitional meetings involving paediatric and adult team, then welcomed in specialized consultations for adolescents with “passport”	In year before transition: 10.49% (1.68); in year after transition: 9.55% (1.82)	75% of participants benefited from this program and demonstrated an improvement in their metabolic control the year following transition to adult care service	–
Farrell et al.,^30^ 2018, Australia, Audit of records	1	439	245 (55.8)	Median age (IQR): 18 (17–19)	Clinic model that ensures a transition coordinator arranging booking, SMS text reminders, access to multidisciplinary team, phone support for sick days	Median HbA_1c_ (IQR): at first appointment: 8.5% (7.6%–10.2%); at 18‐month f‐up 8.6% (7.7%–10.2%); at 30‐month f‐up 8.7% (7.7%–10.0%)	The median HbA_1c_ at the first appointment did not change at the 18‐month f‐up or at the 30‐month f‐up: continuing engagement with the multidisciplinary transition service prevented deterioration in HbA_1c_ following transition	–
Kapellen et al.,^35^ 2018, Germany, Retrospective (database extracted data)	1	1283	711 (55.4)	21.5 (5.1)	–	Last year paediatric: 8.95% (2.04); 2 years before transfer: 8.71% (2.05) (in 1043 documented cases); first year of adult treatment: 9.20% (2.34); second year after transfer: 8.81% (2.14)	Mean HbA_1c_ increase in first year of adult treatment of 0.25% (significantly higher) then in the second year decreased to 8.81%	–
Weigensberg et al.,^28^ 2018, USA, Pilot intervention study (non‐randomized)	1 and 2	37 (9 in DEC; 28 in control group)	5 (55.6) in DEC; 13 (46.4) in control group	19.78 (1.09) in DEC; 19.54 (1.00) in control group	DEC: a 12‐week multimodality, holistic, facilitated group intervention consisting of ‘council’ process based on indigenous community practices, stress reduction guided imagery, narrative medicine modalities and other integrative modalities	DEC group: baseline (*n* = 9) 10.54% (2.51); 6 months (*n* = 9) 9.98 (2.58)%; 12 months (*n* = 9) 9.89% (2.74); Control group: baseline (*n* = 28) 9.15% (2.00); 6 months (*n* = 24) 9.00% (1.79); 12 months (*n* = 22) 8.98% (2.05)	Modest difference in reduction in HbA_1c_ in the DEC group compared with the non‐DEC, but not significant	

Abbreviations: CSSI, continuous subcutaneous insulin infusion; DEC, Diabetes Empowerment Council; ED, emergency department; f‐up, follow‐up; HbA_1c_, glycated haemoglobin; HI‐ATT, high attention to transitional care; LO‐ATT, low attention to transitional care; RF, risk factors; RSG, Ready Steady Go; SD, standard deviation; Y, years.

The transition programs analysed in the included studies varied and included:

- A semi‐structured program involving an initial meeting with an adult diabetologist prior to transition.^12^
- The ‘Ready Steady Go’ (RSG) holistic structured transition program: a comprehensive approach to transitioning focusing on the empowerment of the young people to equip them with the necessary skills.^22^
- Comparison of more (HI‐ATT) versus less attention (LO‐ATT) to transitional care.^23^
- A comparison between a control group treated with usual care and the intervention group which received usual care plus support from a transition coordinator (using communication technology).^24^
- A diabetes transition clinic, with a multidisciplinary team involved in the process of transition (including endocrinologist, educator and dietitian) with one of the educators serving as a coordinator.^25^
- A comparison between standard care versus a transition programme with the central figure of a transition coordinator who maintains contact with participants between the visits and facilitates support to people with diabetes, with the intervention lasting 18 months (6 in paediatric and 12 in adult care).^26^
- A structured program of transition with transitional meetings including paediatric and adult specialists; then, when children were ready, they were welcomed in specialized consultations for AYA with a unique ‘passport’.^27^
- The diabetes Empowerment Council (DEC): a 12‐week multimodality, holistic, facilitated group intervention, comprising a ‘council’ procedure grounded in indigenous community customs, guided imagery for stress reduction and other integrative techniques, involving also narrative medicine techniques.^28^
- A therapeutic care and education program in 4 phases (12 months): a transfer process coordinated from paediatric and adult units, and individual, group and telematic visits.^29^
- A clinic model that ensures a transition coordinator/diabetes educator arranging booking and rebooking, SMS text reminders, access to a multidisciplinary team and phone support for sick days.^30^
- A structured transition programme designed around a receivership model, with care coordination led by the adult endocrinology team to support AYA (age 18–23) through the transition process while working closely with the paediatric endocrinology team. Central to the programme redesign is a transition orientation session, which was a 2.5‐h group class for young adults and their support person(s). Other key elements of the programme redesign included the assessment of transition readiness, the use of a transition coordinator and transition meetings between paediatric and adult endocrinologists to discuss patient‐specific concerns and workflows and to facilitate care coordination.^31^

Of the eleven studies that evaluated a structured or semi‐structured transition program, two^25^, ^27^ reported a reduction in HbA_1c_ levels after transition indicating improved glycaemic control, while eight^12^, ^22^, ^23^, ^24^, ^26^, ^28^, ^29^, ^30^ found no significant difference in HbA_1c_ levels. The study by Iyengar et al.^31^ showed a decrement of HbA_1c_ values after transition for AYA with type 1 diabetes and no significant difference for AYA with type 2 diabetes.

Among the nine papers that did not examine a specific program, four noticed an increase in HbA_1c_ levels post‐transition, indicating worsening of metabolic control,^32^, ^33^, ^34^, ^35^ while five found no significant difference in the HbA_1c_ levels post‐transition.^36^, ^37^, ^38^, ^39^, ^40^ In particular, the study by Soliman et al.^37^ showed that transitioning at an older age was not significantly associated with HbA_1c_ levels but did correlate with other clinical outcomes such as Emergency Department visits. The paper by Tilden et al.^38^ found no significant difference in the values of HbA_1c_ pre‐ and post‐transition but did report a slight increase in mean HbA_1c_ for every six‐month delay between the last paediatric visit and the first adult visit.

Considering other factors influencing transition, the study by Sritharan et al.^25^ highlighted that those with, compared with those without a mental health condition, had higher HbA_1c_. According to Ali et al.,^39^ although co‐location of paediatric and transition clinics improved medical engagement, this did not equate to better glycaemic control or complication rates.

### 3.2 Studies quality

The quality of the studies included assessed using the ROBINS‐I tool is shown in the Figure S1. Four^12^, ^27^, ^28^, ^39^ out of twenty studies showed a high risk of bias in at least one domain: The most common issues contributing to this high risk were a lack of control for confounders in the analysis, inadequate allocation concealment and lack of blinding. Despite these issues, the majority of the studies included were judged to have an overall good quality with a low risk of bias.

### 3.3 Meta‐analysis results and quantitative synthesis

**FIGURE 2:**
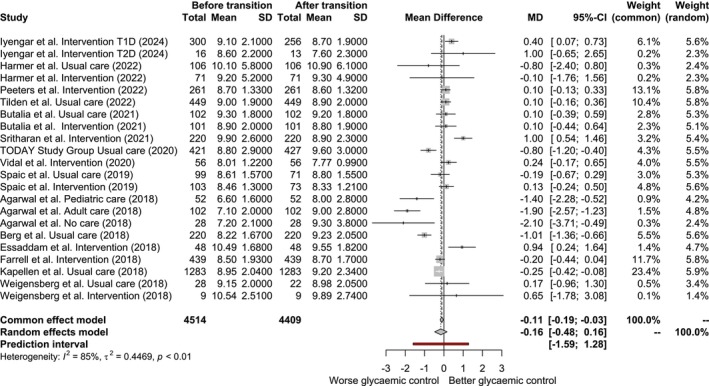
Forest plot of meta‐analysis: Mean difference in HbA_1c_ levels pre‐ and post‐transition. HbA_1c_ values are reported here in percentage (%) according to the Diabetes Control and Complications Trial (DCCT) units for clarity of the figure. These values can be converted to International Federation of Clinical Chemistry and Laboratory Medicine (IFCC) units (mmol/mol) using the official IFCC conversion tables.

The meta‐analysis results are illustrated in the forest plot shown in Figure 2, which presents the mean difference in HbA_1c_ levels pre‐ and post‐transition for every study included. A priori, we selected the random effects model (REM) due to the heterogeneity among studies. The REM estimated a mean difference (MD) of −1.75 mmol/mol (−0.16%) [95% CI −5.24 – 1.75 mmol/mol (−0.48%–0.16%)], where negative values indicate higher HbA_1c_ post‐transition, suggesting that HbA_1c_ levels were, on average, 1.75 mmol/mol (0.16%) higher post‐transition than pretransition; however, this result was not statistically significant.

For reference, the fixed effects model (FEM) estimated a MD of −1.20 mmol/mol (−0.11%) [95% CI –2.08 to –0.33 mmol/mol (−0.19% to –0.03%)], indicating a slight but statistically significant worsening in glycaemic control post‐transition. However, given the substantial heterogeneity among studies, we relied on the REM results as the primary estimate.

The funnel plot from the meta‐analysis reveals an imbalance in study distribution, with fewer small studies at the bottom and more large studies at the top. A slight asymmetry is observed, as a greater number of studies report a negative mean difference, indicating worsening glycaemic control post‐transition. However, Egger's test yielded a *p*‐value of 0.813, which is not statistically significant, suggesting no strong evidence of publication bias (Figure S2).

### 3.4 Subgroup analysis to explore heterogeneity

**FIGURE 3:**
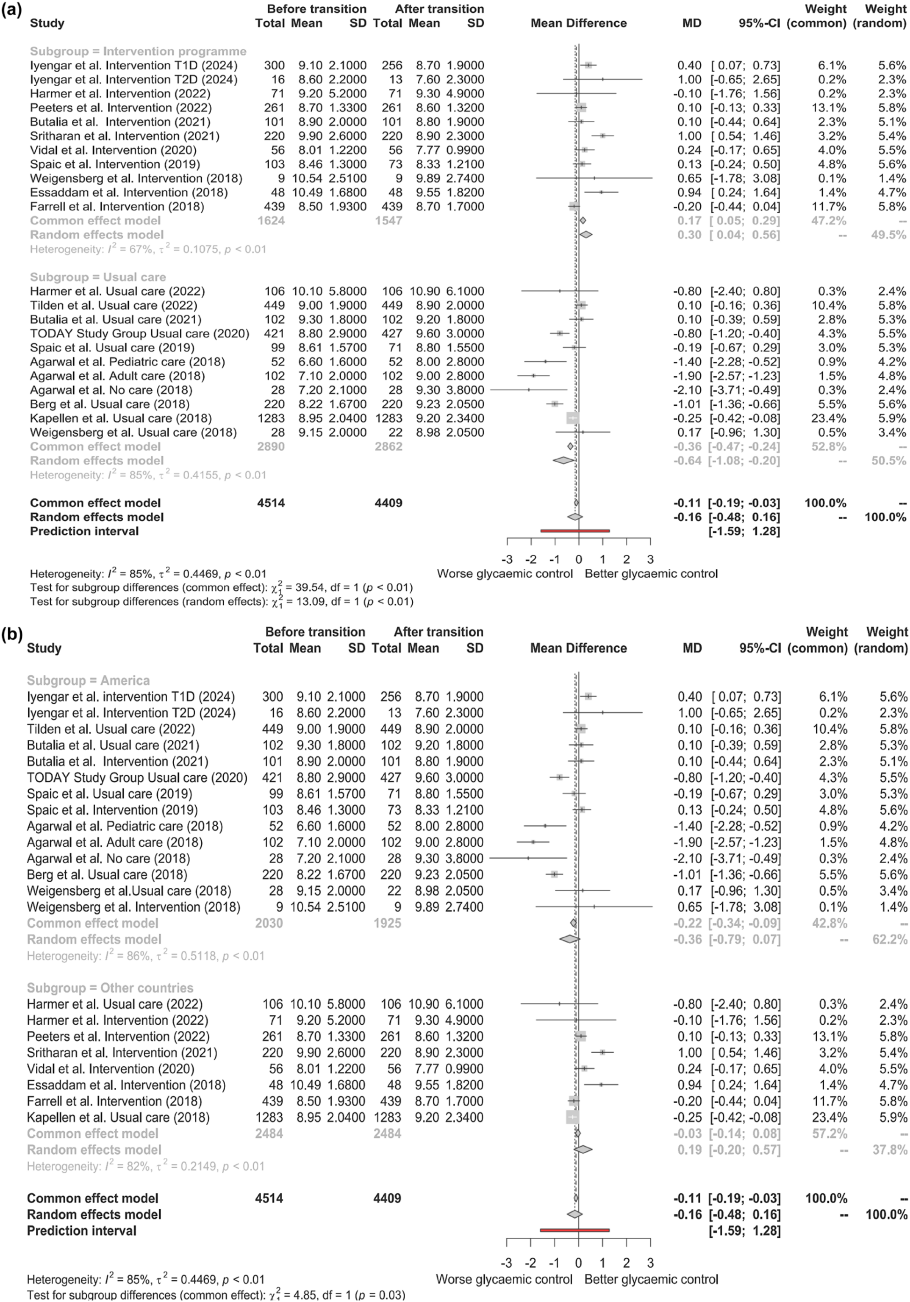
Forest plot of subgroup analysis: Panel a, impact of transition programs on HbA_1c_ levels; Panel b, HbA_1c_ changes by geographic region. HbA_1c_ values are reported here in percentage (%) according to the Diabetes Control and Complications Trial (DCCT) units for clarity of the figure. These values can be converted to International Federation of Clinical Chemistry and Laboratory Medicine (IFCC) units (mmol/mol) using the official IFCC conversion tables.

Heterogeneity among the included studies was high, with an *I* ^2^ value of 85%, indicating substantial variability across studies, which may limit the interpretability of the overall meta‐analysis results. To explore the substantial heterogeneity, we performed a subgroups analysis. As shown in Figure 3 Panel A, studies were stratified into two groups: those implementing intervention programs and those following usual care, each represented by separate forest plots.

The heterogeneity among the studies implementing intervention programs was 67%, indicating moderate‐to‐high heterogeneity within this subgroup. The MD in the REM was 3.28 mmol/mol (0.30%) [95% CI 0.44–6.12 mmol/mol (0.04%–0.56%)], which was statistically significant. This suggests that the intervention programs may contribute to better glycaemic control post‐transition.

On the contrary, the heterogeneity among studies without an intervention program was 85%, and the MD estimated was −6.99 mmol/mol (−0.64%) [95% CI –11.79 to –2.19 mmol/mol (−1.08% to –0.20%)], indicating a statistically significant worsening of glycaemic control. This suggests that the absence of a structured transition program negatively impacts HbA_1c_ levels post‐transition.

We performed a further subgroups analysis, stratifying the studies based on geographic location. Studies were categorized into two groups: seven studies conducted in America (USA and Canada) and eight studies conducted in other regions, resulting in two separate forest plots for comparison (Figure 3 Panel B).

The heterogeneity among the studies conducted in America was 86%, indicating substantial variability within this subgroup. The MD for this subgroup was −3.93 mmol/mol (−0.36%) [95% CI –8.63 to 0.77 mmol/mol (−0.79% to 0.07%)], which was not statistically significant. Similarly, the heterogeneity among the studies conducted outside North America was 82%, indicating high variability. The MD in this subgroup was 2.08 mmol/mol (0.19%) [95% CI –2.19 to 6.23 mmol/mol (−0.20% to 0.57%)], which was also not statistically significant. These findings suggest that the geographic location of the studies does not significantly impact HbA_1c_ levels post‐transition. The high heterogeneity in both subgroups indicates that other factors may contribute more substantially to the observed differences in glycaemic control.

## 4 DISCUSSION

This systematic review and meta‐analysis evaluated the impact of transition from paediatric to adult care on healthcare outcomes, focusing primarily on glycaemic control measured by HbA_1c_ levels. Our findings highlight that structured transition programs showed a significant improvement in glycaemic control. These programs appear to support better diabetes management during the vulnerable transition phase. However, the results highlight a fragmented situation with significant variability across studies. While our analysis focused on HbA_1c_, it is important to consider that differences in healthcare systems, policies and transition programs across countries could play a role. Particularly in countries with fragmented healthcare systems like the United States, where inconsistent insurance coverage may contribute to higher loss to follow‐up and poorer outcomes during the transition period from adolescence to adult care, this may partially explain these disparities, though our study was not designed to directly assess system‐level effects. Further research is needed to explore this question in greater detail.

The role of age at transition remains unclear, with limited research available. One study^37^ reported that transition at an older age was not significantly associated with worse control, but further research is required to clarify its impact.

The meta‐analysis results indicate high heterogeneity across studies, emphasizing the challenges in drawing generalized conclusions. Researchers must consider this when interpreting results and should strive for standardized protocols and outcome measures in future studies to enhance comparability. The REM, which accounts for variability, showed a non‐significant MD of −1.75 mmol/mol (−0.16%) [95% CI –5.24 to 1.75 mmol/mol (−0.48% to 0.16%)], indicating a potential worsening of glycaemic control. Factors contributing to heterogeneity include variations in study settings, healthcare regulations, study populations and designs. Subgroup analyses, one of the most common strategies to explore heterogeneity,^41^ reduced but did not eliminate heterogeneity, underscoring the need for further research (including meta‐regression or sensitivity analyses): They showed a significant improvement of HbA_1c_ for the transition programs group and a significant worsening for the usual care group.

Strength of this study is that it included a substantial number of studies and focused on recent evidence from global literature. However, there are also limitations: Studies included were not homogeneous in the countries where they were conducted, reflecting diverse healthcare systems and organizational structures that may contribute to variability in outcomes, nor in design; the decision to include only studies published from 2018 onward ensured an analysis of the most recent findings but may have excluded relevant older studies. We acknowledge that some studies (e.g. retrospective cohort studies) might not perfectly fit the ROBINS‐I framework. However, for the sake of consistency and comparability across all included studies, we decided to use a single risk of bias tool rather than applying different tools for different study designs, which would have complicated the comparison of the results. The relatively small number of included studies is a result of our strict inclusion criteria focusing specifically on metabolic control and HbA_1c_ levels before and after transition. Furthermore, other relevant outcomes, such as time in range and quality of life, were not consistently reported across studies and therefore should be prioritized in future research.

Including studies with various designs poses challenges for synthesizing evidence: Combining results from different methodologies can introduce inconsistencies. The lack of randomized controlled trials (RCTs) in this review limits the strength of the conclusions. Well‐designed RCTs are needed to provide more robust evidence on the effectiveness of transition programmes in maintaining glycaemic control: Future research should prioritize RCTs using standardized transition programmes with clear outcome measures such as HbA_1c_, diabetic ketoacidosis rates, hypoglycaemia, time in range and quality of life.

Most research on glycaemic control during healthcare transition focuses on type 1 diabetes, with limited data on type 2 diabetes; in fact, only 5 of the studies included analysed data of AYA with type 2 diabetes.^25^, ^28^, ^31^, ^32^, ^33^ This imbalance may lead to an incomplete understanding of the transition‐related challenges for people with type 2 diabetes. Given the rising prevalence of type 2 diabetes,^42^ it is crucial to include this population in future studies.

To address the challenges in maintaining optimal glycaemic control during the transition from paediatric to adult care, hospitals and clinics have developed various transition programmes aimed at enhancing healthcare continuity during this crucial phase. According to the findings of this study, transition programmes showed a significant improvement in glycaemic control and therefore may help maintain or improve glycaemic control during this phase and/or prevent its decline. This must be interpreted with caution due to limited long‐term follow‐up. Many studies focus on short‐term outcomes, leaving a significant gap in understanding how these programmes affect AYA with diabetes over several years. Furthermore, transition programmes often include multiple components, making it difficult to determine which elements are most effective. Developing public health guidelines is becoming challenging, as transition programmes are complex health interventions. The WHO has recently worked on methods to improve evidence synthesis and develop impactful guidelines, taking into account the diverse needs of stakeholders, from healthcare providers to individuals, to implement interventions within complex health systems.^42^ Among the studies included in this review, those that reported improved glycaemic control post‐transition shared several key features within their structured transition programmes. First, the presence of a dedicated transition coordinator appeared to be a critical component. In one study, care coordination was led by the adult endocrinology team and included regular assessment of transition readiness, suggesting that proactive preparation and early engagement with adult services support a smoother transition process. Another effective programme incorporated joint transitional meetings involving both paediatric and adult care teams, followed by targeted consultations for adolescents using tools such as a ‘transition passport’. A third successful model included a multidisciplinary diabetes transition clinic with an endocrinologist, educator and dietitian, where one of the educators served as a coordinator, ensuring continuity and personalized care. This extra attention and support may be helpful in keeping AYA engaged in care and preparing them for adult care.^13^ Additionally, psychosocial support is essential, particularly for AYA with pre‐existing mental health conditions, as unmanaged stressors can exacerbate glycaemic instability.^25^ A good communication between AYA with diabetes and doctors together with transition readiness, that can be assessed through questionnaires, could play an important role^43^ as well as the use of technology, including mobile applications and social media together with online education, which may support self management and engagement in care during this transition.^44^

In conclusion, these findings suggest that structured communication between paediatric and adult teams, assessment of transition readiness and the presence of a multidisciplinary support system are promising features that may facilitate better glycaemic outcomes. However, further research is needed to establish evidence‐based guidelines. This knowledge could enable healthcare policymakers to prioritize and invest in the most impactful programmes, allowing hospitals to integrate best practices into their transitional care strategies.

## AUTHOR CONTRIBUTIONS

G.D. conceptualized the research, researched and analysed data, wrote the first draft of the manuscript, contributed to the discussion, reviewed and edited the manuscript, designed and edited the graphical abstract. C.M. researched and analysed data and reviewed the manuscript. S.E.H., D.M. and U.S. contributed to the discussion and reviewed the manuscript. M.A. conceptualized the research, analysed data, reviewed and edited the manuscript. All authors approved the final version of the manuscript.

## FUNDING INFORMATION

None.

## CONFLICT OF INTEREST STATEMENT

None.

## STUDY REGISTRATION

The manuscript was registered on PROSPERO with registration number CRD42024515780.

## Supporting information


**Data S1:** Supplementary Information.

## References

[1] Libman I , Haynes A , Lyons S , et al. ISPAD Clinical Practice Consensus Guidelines 2022: definition, epidemiology, and classification of diabetes in children and adolescents. Pediatr Diabetes. 2022;23(8):1160‐1174. doi:10.1111/pedi.13454 36537527

[2] Ogle GD , Wang F , Gregory GA , et al. Type 1 diabetes estimates in children and adults. IDF Diabetes Atlas. Accessed January 25, 2024. https://diabetesatlas.org/atlas/t1d‐index‐2022/

[3] 14. Children and adolescents: standards of care in diabetes—2025. Diabetes Care. American Diabetes Association. Accessed July 15, 2025. https://diabetesjournals.org/care/article/48/Supplement_1/S283/157559/14‐Children‐and‐Adolescents‐Standards‐of‐Care‐in

[4] de Bock M , Codner E , Craig ME , et al. ISPAD Clinical Practice Consensus Guidelines 2022: glycemic targets and glucose monitoring for children, adolescents, and young people with diabetes. Pediatr Diabetes. 2022;23(8):1270‐1276. doi:10.1111/pedi.13455 36537523 PMC10107615

[5] Schultz AT , Smaldone A . Components of interventions that improve transitions to adult care for adolescents with type 1 diabetes. J Adolesc Health. 2017;60(2):133‐146. doi:10.1016/j.jadohealth.2016.10.002 27939878

[6] Rasmussen B , Terkildsen Maindal H , Livingston P , Dunning T , Lorentzen V . Psychosocial factors impacting on life transitions among young adults with type 2 diabetes: an Australian—Danish qualitative study. Scand J Caring Sci. 2016;30(2):320‐329. doi:10.1111/scs.12248 26037014

[7] Garvey KC , Wolpert HA , Laffel LM , Rhodes ET , Wolfsdorf JI , Finkelstein JA . Health care transition in young adults with type 1 diabetes: barriers to timely establishment of adult diabetes care. Endocr Pract. 2013;19(6):946‐952. doi:10.4158/EP13109.OR 23807526 PMC4034180

[8] Lyons SK , Becker DJ , Helgeson VS . Transfer from pediatric to adult health care: effects on diabetes outcomes. Pediatr Diabetes. 2014;15(1):10‐17. doi:10.1111/pedi.12106 24350767 PMC4097315

[9] Wisk LE , Garvey KC , Fu C , Landrum MB , Beaulieu ND , Chien AT . Diabetes‐focused health care utilization among adolescents and young adults with type 1 diabetes. Acad Pediatr. 2024;24(1):59‐67. doi:10.1016/j.acap.2023.05.001 37148967 PMC12310162

[10] GotTransition.org . Got Transition®. GotTransition.org. 2020; Accessed July 4, 2025. https://gottransition.org/

[11] Peters A , Laffel L , the American Diabetes Association Transitions Working Group . Diabetes care for emerging adults: recommendations for transition from pediatric to adult diabetes care systems: a position statement of the American Diabetes Association, with representation by the American College of Osteopathic Family Physicians, the American Academy of Pediatrics, the American Association of Clinical Endocrinologists, the American Osteopathic Association, the Centers for Disease Control and Prevention, Children with Diabetes, The Endocrine Society, the International Society for Pediatric and Adolescent Diabetes, Juvenile Diabetes Research Foundation International, the National Diabetes Education Program, and the Pediatric Endocrine Society (formerly Lawson Wilkins Pediatric Endocrine Society). Diabetes Care. 2011;34(11):2477‐2485. doi:10.2337/dc11-1723 22025785 PMC3198284

[12] Schweizer R , Loesch‐Binder M , Hayn C , et al. Transition from childhood to adult Care in Patients with type 1 diabetes: 20 years of experience from the Tübinger transition study. Exp Clin Endocrinol Diabetes. 2023;131(10):532‐538. doi:10.1055/a-2132-9585 37467782

[13] Olsson S , Otten J , Blusi M , Lundberg E , Hörnsten Å . Experiences of transition to adulthood and transfer to adult care in young adults with type 1 diabetes: a qualitative study. J Adv Nurs. 2023;79:4621‐4634. doi:10.1111/jan.15740 37357405

[14] DeLacey S , Papadakis J , James S , et al. A systematic review of interventions for the transition to adult healthcare for young people with diabetes. Curr Diab Rep. 2025;25(1):21. doi:10.1007/s11892-025-01578-2 39890661 PMC12228552

[15] PRISMA. Accessed January 25, 2024. http://prisma‐statement.org/prismastatement/checklist.aspx

[16] Chapter 25: Assessing risk of bias in a non‐randomized study. Accessed January 25, 2024. https://training.cochrane.org/handbook/current/chapter‐25

[17] McGuinness LA , Higgins JPT . Risk‐of‐bias VISualization (robvis): an R package and shiny web app for visualizing risk‐of‐bias assessments. Res Synth Methods. 2021;12(1):55‐61. doi:10.1002/jrsm.1411 32336025

[18] Hoelzel W , Weykamp C , Jeppsson JO , et al. IFCC reference system for measurement of hemoglobin A1c in human blood and the National Standardization Schemes in the United States, Japan, and Sweden: a method‐comparison study. Clin Chem. 2004;50(1):166‐174. doi:10.1373/clinchem.2003.024802 14709644

[19] Hozo SP , Djulbegovic B , Hozo I . Estimating the mean and variance from the median, range, and the size of a sample. BMC Med Res Methodol. 2005;5:13. doi:10.1186/1471-2288-5-13 15840177 PMC1097734

[20] Egger M , Davey Smith G , Schneider M , Minder C . Bias in meta‐analysis detected by a simple, graphical test. BMJ. 1997;315(7109):629‐634. 9310563 10.1136/bmj.315.7109.629 PMC2127453

[21] Schwarzer G . meta: general package for meta‐analysis. Accessed May 30, 2024. https://cran.r‐project.org/web/packages/meta/index.html

[22] Harmer MJ , Everitt LH , Parker L , Davis N , Connett G , Nagra A . Structured transition is associated with improved outcomes in diabetes. Pract Diab. 2022;39(1):18.

[23] Peeters MAC , Sattoe JNT , Bronner MB , Bal RA , van Staa A . The added value of transition programs in Dutch diabetes care: a controlled evaluation study. J Pediatr Nurs. 2022;62:155‐163. doi:10.1016/j.pedn.2021.08.004 34419327

[24] Butalia S , Crawford SG , McGuire KA , Dyjur DK , Mercer JR , Pacaud D . Improved transition to adult care in youth with type 1 diabetes: a pragmatic clinical trial. Diabetologia. 2021;64(4):758‐766. doi:10.1007/s00125-020-05368-1 33439284

[25] Sritharan A , Osuagwu UL , Ratnaweera M , Simmons D . Eight‐year retrospective study of young adults in a diabetes transition clinic. Int J Environ Res Public Health. 2021;18(23):12667. doi:10.3390/ijerph182312667 34886392 PMC8656842

[26] Spaic T , Robinson T , Goldbloom E , et al. Closing the gap: results of the multicenter Canadian randomized controlled trial of structured transition in young adults with type 1 diabetes. Diabetes Care. 2019;42(6):1018‐1026. doi:10.2337/dc18-2187 31010873

[27] Essaddam L , Kallali W , Jemel M , et al. Implementation of effective transition from pediatric to adult diabetes care: epidemiological and clinical characteristics—a pioneering experience in North Africa. Acta Diabetol. 2018;55(11):1163‐1169. doi:10.1007/s00592-018-1196-x 30074090

[28] Weigensberg MJ , Vigen C , Sequeira P , et al. Diabetes empowerment council: integrative pilot intervention for transitioning young adults with type 1 diabetes. Glob Adv Health Med. 2018;7:2164956118761808. doi:10.1177/2164956118761808 29552422 PMC5846920

[29] Vidal M , Jansà M , Roca D , et al. Hypoglycaemia unawareness in young people with type 1 diabetes transferred to an adult center. Endocrinol Diabetes Nutr. 2020;67(6):394‐400. doi:10.1016/j.endinu.2019.07.009 31668682

[30] Farrell K , Fernandez R , Salamonson Y , Griffiths R , Holmes‐Walker DJ . Health outcomes for youth with type 1 diabetes at 18 months and 30 months post transition from pediatric to adult care. Diabetes Res Clin Pract. 2018;139:163‐169. doi:10.1016/j.diabres.2018.03.013 29534994

[31] Iyengar JJ , Ang L , Rodeman KB , et al. A novel receivership model for transition of young adults with diabetes: experience from a single‐center academic transition program. Endocr Pract. 2024;30(2):113‐121. doi:10.1016/j.eprac.2023.11.008 38029926 PMC11913126

[32] Health care coverage and glycemic control in young adults with youth‐onset type 2 diabetes: results from the TODAY2 study. Diabetes Care. 2020;43(10):2469‐2477. doi:10.2337/dc20-0760 32778555 PMC7510035

[33] Agarwal S , Raymond JK , Isom S , et al. Transfer from paediatric to adult care for young adults with type 2 diabetes: the SEARCH for diabetes in youth study. Diabet Med. 2018;35(4):504‐512. doi:10.1111/dme.13589 29377258 PMC6130201

[34] Berg CA , Wiebe DJ , Suchy Y , et al. Executive function predicting longitudinal change in type 1 diabetes management during the transition to emerging adulthood. Diabetes Care. 2018;41(11):2281‐2288. doi:10.2337/dc18-0351 30131398 PMC6196825

[35] Kapellen TM , Müther S , Schwandt A , et al. Transition to adult diabetes care in Germany‐high risk for acute complications and declining metabolic control during the transition phase. Pediatr Diabetes. 2018;19(6):1094‐1099. doi:10.1111/pedi.12687 29691964

[36] Hodnekvam K , Iversen HH , Gani O , Brunborg C , Skrivarhaug T . Do adolescents and emerging adults receive the diabetes care they truly need? A nationwide study of the quality of diabetes health care during the transition from paediatric to adult care. Diabet Med. 2023;40(7):e15091. doi:10.1111/dme.15091 36932850

[37] Soliman D , Crowley MJ , Manning A , et al. Transition from pediatric to adult care in type 1 diabetes mellitus: a longitudinal analysis of age at transfer and gap in care. BMJ Open Diabetes Res Care. 2022;10(6):e002937. doi:10.1136/bmjdrc-2022-002937 PMC9639054 36328375

[38] Tilden DR , French B , Shoemaker AH , Corathers S , Jaser SS . Prolonged lapses between pediatric and adult care are associated with rise in HbA1c and inpatient days among patients with type 1 diabetes. Diabetes Res Clin Pract. 2022;192:110113. doi:10.1016/j.diabres.2022.110113 36208847 PMC9867942

[39] Ali N . Adolescent with type 1 diabetes attending a metropolitean hospital transition clinic—a single centre experience. J Paediatr Child Health. 2021;57(Suppl 1):16. doi:10.1111/jpc.15466

[40] Walch AM , Cobb CE , Tsaih SW , Cabrera SM . The medical transition of young adults with type 1 diabetes (T1D): a retrospective chart review identifies areas in need of improvement. Int J Pediatr Endocrinol. 2020;2020:10. doi:10.1186/s13633-020-00080-8 32514267 PMC7254679

[41] Higgins JPT , López‐López JA , Becker BJ , et al. Synthesising quantitative evidence in systematic reviews of complex health interventions. BMJ Glob Health. 2019;4(Suppl 1):e000858. doi:10.1136/bmjgh-2018-000858 PMC6350707 30775014

[42] Khan MAB , Hashim MJ , King JK , Govender RD , Mustafa H , Al Kaabi J . Epidemiology of type 2 diabetes—global burden of disease and forecasted trends. J Epidemiol Glob Health. 2020;10(1):107‐111. doi:10.2991/jegh.k.191028.001 32175717 PMC7310804

[43] Baker AC , Wiebe DJ , Kelly CS , et al. Structural model of patient‐centered communication and diabetes management in early emerging adults at the transfer to adult care. J Behav Med. 2019;42(5):831‐841. doi:10.1007/s10865-019-00012-9 30680592 PMC6656634

[44] Esposito S , Rosafio C , Antodaro F , et al. Use of telemedicine healthcare systems in children and adolescents with chronic disease or in transition stages of life: consensus document of the Italian Society of Telemedicine (SIT), of the Italian Society of Preventive and Social Pediatrics (SIPPS), of the Italian Society of Pediatric Primary Care (SICuPP), of the Italian Federation of Pediatric Doctors (FIMP) and of the Syndicate of Family Pediatrician Doctors (SIMPeF). J Pers Med. 2023;13(2):235. doi:10.3390/jpm13020235 36836469 PMC9965862

