# Supplementary material for: Quality of diabetes mellitus healthcare and metabolic control during transition from paediatric to adult care: A systematic review and meta‐analysis
Source: Diabet Med. 2025 Aug 20;42(10):e70125. doi: 10.1111/dme.70125 (PMC12434428; doi:10.1111/dme.70125)

#### **Supplementary material**

**Tables**

Table S1……………………………………………………………………………….…..…………2

**Figures**

Figure S1………………………………………………………………………………………....….3

Figure S2………………………………………………………………………………………..……4

**Table S1.** PICO Framework

| *Population* | Adolescents and young adults with diabetes (type 1 or type 2 diabetes) who transitioned from children healthcare to adult healthcare |
| --- | --- |
| *Intervention* | Specific transition programs for adolescents and young adults with diabetes from childhood to young adulthood |
| *Comparators* | Standardized programs for transition of care for young adults with diabetes |
| *Outcomes* | Quality of diabetes care by measuring the glycated haemoglobin values before and after transition |

**Figure S1.** Risk of bias assessment

*
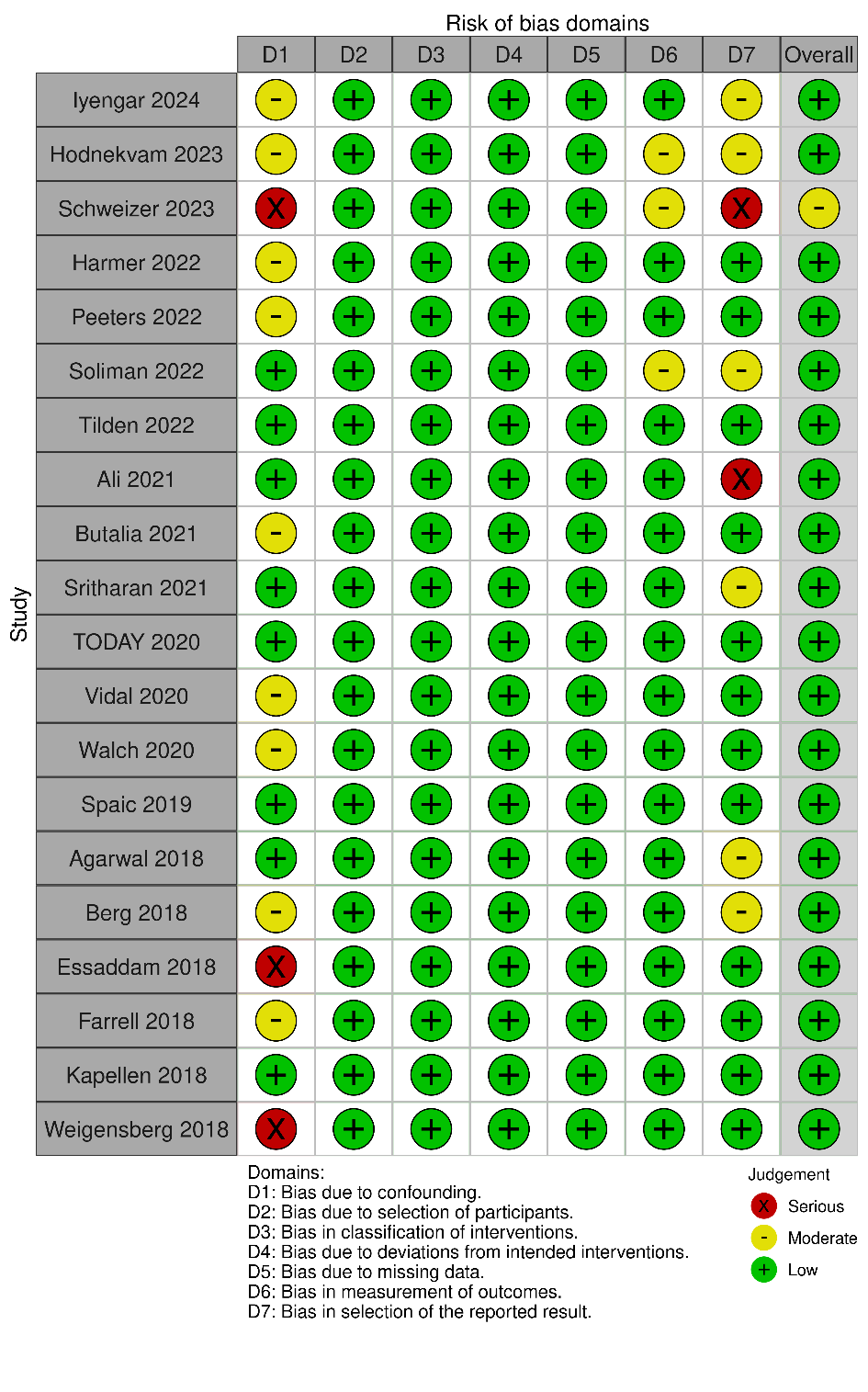
*

**Figure S2.** Funnel plot for publication bias assessment


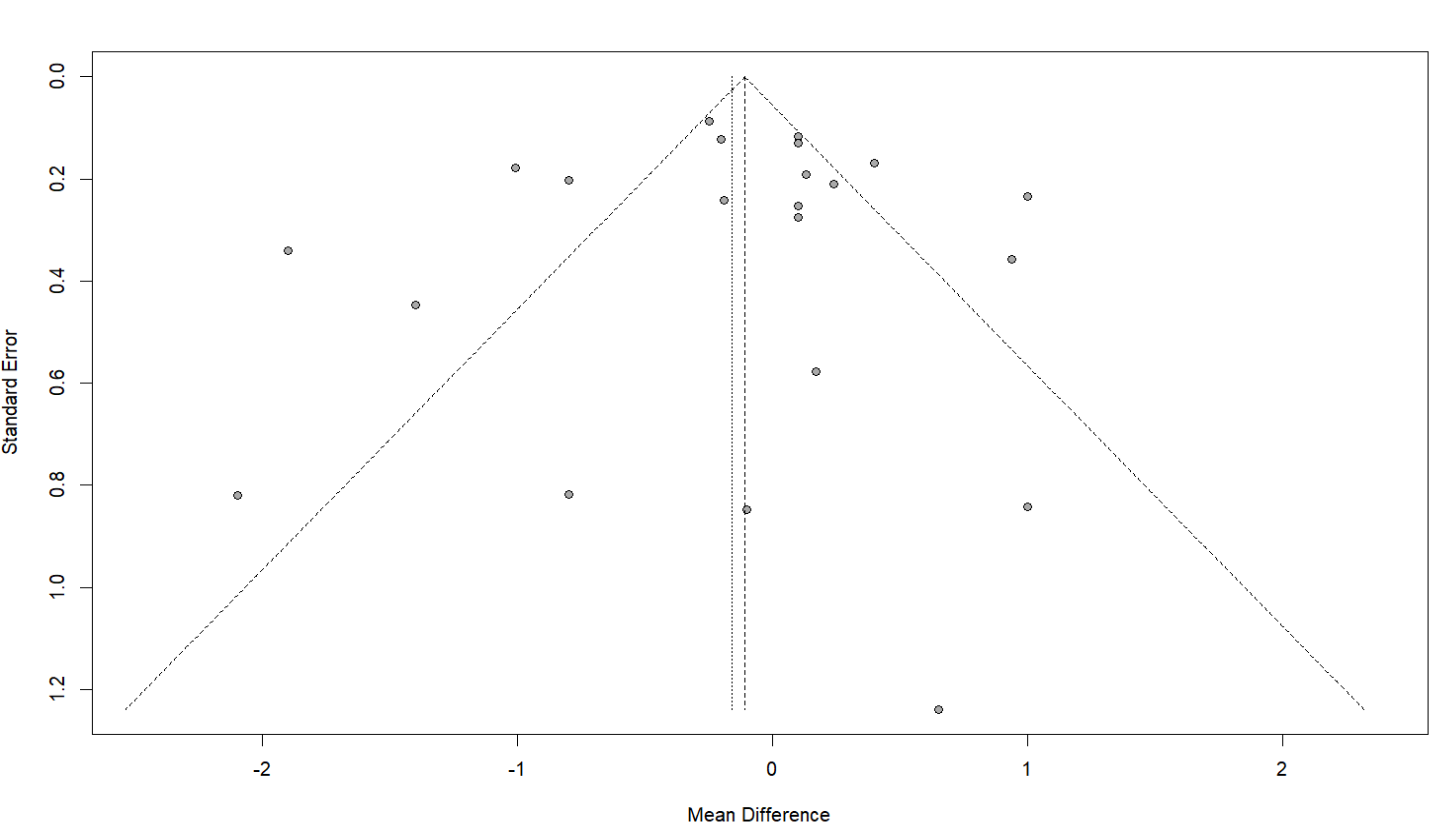

Supplement: Supplementary file 1 — Data S1: Supplementary Information. [file DME-42-e70125-s001.docx]
